# Improving the Angular Velocity Measured with a Low-Cost Magnetic Rotary Encoder Attached to a Brushed DC Motor by Compensating Magnet and Hall-Effect Sensor Misalignments

**DOI:** 10.3390/s21144763

**Published:** 2021-07-12

**Authors:** Jordi Palacín, David Martínez

**Affiliations:** Laboratory of Robotics, Universitat de Lleida, Jaume II 69, 25001 Lleida, Spain; david.martinez@udl.cat

**Keywords:** hall-effect sensor, low-cost brushed DC motor, misalignment correction

## Abstract

This paper proposes a method to improve the angular velocity measured by a low-cost magnetic rotary encoder attached to a brushed direct current (DC) motor. The low-cost magnetic rotary encoder used in brushed DC motors use to have a small magnetic ring attached to the rotational axis and one or more fixed Hall-effect sensors next to the magnet. Then, the Hall-effect sensors provide digital pulses with a duration and frequency proportional to the angular rotational velocity of the shaft of the encoder. The drawback of this mass produced rotary encoder is that any structural misalignment between the rotating magnetic field and the Hall-effect sensors produces asymmetric pulses that reduces the precision of the estimation of the angular velocity. The hypothesis of this paper is that the information provided by this low-cost magnetic rotary encoder can be processed and improved in order to obtain an accurate and precise estimation of the angular rotational velocity. The methodology proposed has been validated in four compact motorizations obtaining a reduction in the ripple of the estimation of the angular rotational velocity of: 4.93%, 59.43%, 76.49%, and 86.75%. This improvement has the advantage that it does not add time delays and does not increases the overall cost of the rotary encoder. These results showed the real dimension of this structural misalignment problem and the great improvement in precision that can be achieved.

## 1. Introduction

Most control systems used in automation and robotics require the measurement of the angular rotational velocity of a motor using a sensorless approach [1], using internal sensors [2,3], or using external rotary encoders [4]. The low-cost standard defined by the industry is an external rotary encoder based on a rotating magnet ring with two, four, six, or more alternating poles attached to the rotating shaft and one, two, or three fixed Hall-effect digital sensors. The magnetic changes originated by the rotation of the magnetic ring is then detected by the Hall-effect sensors and converted into digital pulses that can be used to estimate the angular velocity of the shaft. Then, the accuracy and precision of the angular velocity measured lies on the symmetry of the signals generated by the encoder that depends on the perfect alignment between the poles of the magnet and the Hall-effect sensors.

The analysis of the effects of magnetic misalignments has been deeply studied in the case of brushless DC (BLDC) motors because this motor requires an activation sequence with a precise phase difference of 120 angular degrees that is usually detected by using internal Hall-effect sensors placed strategically in the motor. Alaeinovin et al. [5] demonstrated the existence of misalignments in the Hall-effect sensors used in BLDC motors and analyzed the influence of this misalignments on the stator current and torque in the case of medium and low-precision motors. Hung et al. [6] proposed the calculation of the time difference between two rising/falling edges of the signals generated by the Hall-effect sensors as a method to accurately estimate the angle between two Hall-effect sensors in a BLDC motor. This method was proved very effective in order to reduce the errors on low-resolutions position feedbacks. Based on this approach, Samoylenko et al. [7] analyzed the misalignment of Hall sensors in BLDC motors and proposed a moving-average filtering technique applied on the Hall Effect signals in order to mitigate the influence of unbalanced sensors on the performance of the motor. Lately, Alaeinovin et al. [8] proposes filtering the Hall-sensor signals of BLDC motors in order to improve the driving performance. However, the application of a filtering strategy was used to have the drawback of adding a time delay on the information gathered.

In a different approach, Beccue et al. [9] proposed a Hall Effect position observer for the mitigation of ripple in BLDC motors. In this case the observer includes an automated routine to compensate the misalignment of Hall sensors based on computing the time between Hall-Effect transitions and compare this value with the ideal values stored in a lookup table. Similarly, Ortiz et al. [10] simulated a method for high resolution position estimation of a BLDC motor based on the measurement of the pulses obtained from three Hall Effect sensors providing 12 rising and falling edges or 12 pulses per revolution while Alcázar et al. [11] avoids possible asymmetries originated by the Hall Effect sensor by using only the rising edges of the output signal.

In the one side, this paper is inspired in the contribution of Hung et al. [12] that proposed a method for correcting the errors occurring during the measurement of the angular rotational velocity of a BLDC motor. This method determines the degree of misalignment of the internal Hall-effect sensors and proposes a corrective ratio to reduce the effect of this misalignment in the estimation of the angular rotational velocity of on a two pole BLDC motor. On the other side, this paper is also inspired in the contribution of Kolano et al. [13] that proposes a method for determining the mechanical position of the shaft of a BLDC with more than one pole pair. This method is based on the analysis of the distribution of the errors obtained when measuring the angular rotational velocity with the internal Hall-effect sensors relative to an external encoder in an open loop drive system. The conclusion was that the proposed method can correct the inaccurate fixing of the Hall-effect sensors in the BLDC. More information about this specific problem can be found in the comprehensive review of Paredes et al. [14], focused on the application of magnetic encoders as position sensors in BLDC motors.

### New Contribution

As cited in the introduction, the effect of magnetic misalignment has been studied in depth in the case of BLDC motors, because this misalignment affects directly the performances of the motor. However, this misalignment effect has not been properly analyzed in another application context such as low-cost magnetic rotary encoders used in brushed DC motors. This paper states and analyzes this problem and proposes a method to improve the estimation of the angular rotational velocity. This compact motorization is commonly used to drive the wheels of small mobile robots where the encoder is used provide feedback of the angular rotational velocity of the DC motor and to estimate also the odometry of the mobile robot.

The low-cost magnetic rotary encoder analyzed in this paper is a compact device with a small magnet ring attached to the rotating shaft and one or more fixed Hall-effect sensors placed around the magnet. The rotation of the shaft makes the Hall-effect sensors provide digital pulses with a duration and frequency proportional to the angular rotational velocity of the shaft. Then, any structural misalignment between the magnetic ring and Hall-effect sensors will affect the precision of the measurement.

The new contribution of this paper states this case specific problem, proposes a calibration procedure in order to obtain the misalignment correction coefficients that can compensate the structural misalignment between the rotating magnet and the Hall-effect sensors, and finally proposes a correction procedure to improve the precision of the estimate of the angular rotational velocity. This improvement has the advantage that it does not add time delays in the corrected estimation of the angular rotational velocity.

## 2. Materials and Methods

The materials and methods used in this paper are four compact brushed DC motors that includes a low-cost magnetic encoder, a motor control board, and an input capture method to measure the signal generated by the encoders.

### 2.1. Brushed DC Motor with a Low-Cost Magnetic Encoder

**Figure 1:**
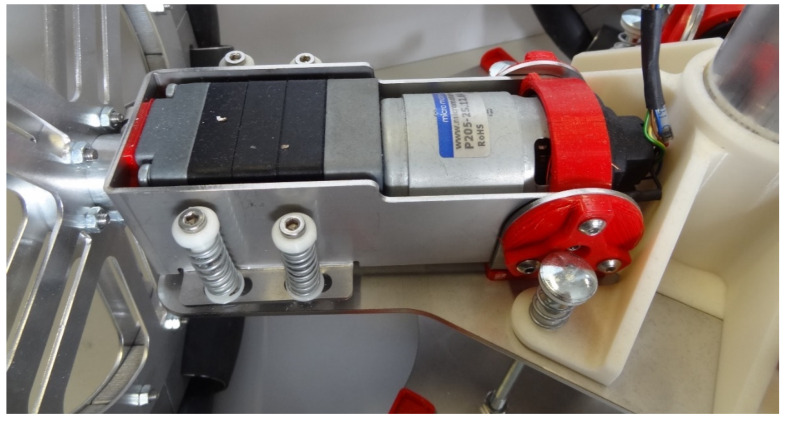
Detail of the compact motorization P205-2S.12.64 that includes a brushed DC motor, a 1:64 gear (at the front), and a six-pole low-cost magnetic encoder (at the back).

The brushed DC motor used in this paper are four units of the DC compact motor: P205-2S.12.64 (145.5 mm), manufactured by Micro Motors SRL (Verderio, Italy) [15]. This motorization includes a brushed DC motor, a 1:64 planetary gearbox (in the front) and a low-cost magnetic quadrature encoder (in the back) directly attached to the motor shaft. The motor operates at 12 V and the gearbox can generate 6 Nm with a power consumption of 57.6 W. Figure 1 shows an image of this compact motor that has been used intensively by the authors in the motion system of several mobile robots [16,17,18,19].

**Figure 2:**
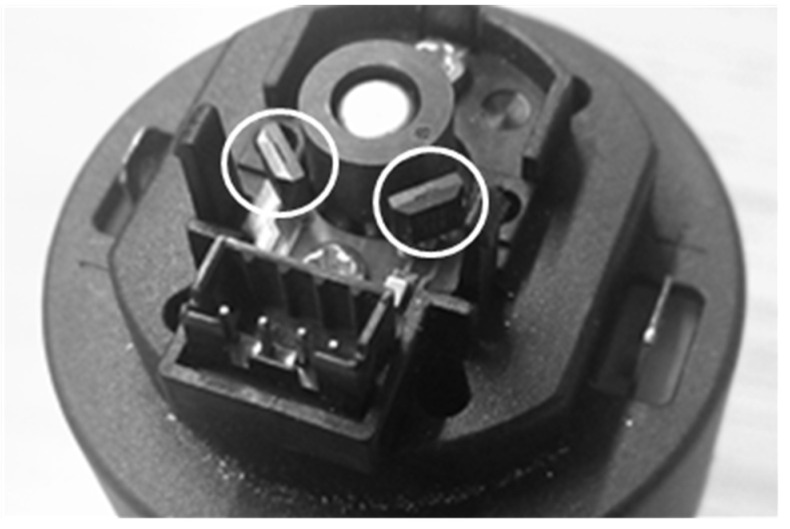
Detail of the low-cost magnetic encoder showing the magnetic ring attached to the motor shaft and the two Hall-Effect sensors (white circle).

Figure 2 shows a detailed image of the low-cost magnetic encoder of this compact motor. The encoder has a six poles magnetic ring and two Hall-Effect sensors (U18 sensor from Unisonic Technologies Co., Ltd., Taipei, Taiwan). This Hall-Effect sensor has an internal electronic circuit which includes a voltage regulator, a quadratic Hall voltage generator, a temperature stability circuit, a signal chopper stabilized amplifier, a Schmitt trigger digital circuit, and an open drain MOSFET as digital output. The on-board regulator allows supply voltages of 3.5 V to 24 V and the output MOSFET can sink up to 20 mA. The Hall-effect sensors are located at a phase of 90° in order to generate a quadrature signal which enables to know both the velocity and direction of rotation. Finally, each Hall-Effect sensor generates three pulses per motor turn as a consequence of the rotation of the six poles magnet.

### 2.2. Motor Control Board

The motor control board used in this paper is based on the STM32F407VGT6 microcontroller from STMicroelectronics (Geneva, Switzerland), a high-performance ARM (Cambridge, UK) Cortex-M4 32-bit RISC core. This microcontroller operates at an internal frequency of 168 MHz with 210 DMIPS and a power dissipation of 139 mW. This microcontroller has an internal SRAM memory of 196 Kb, 1 Mb Flash memory, and includes many internal peripherals for motor control such as timers, PWM generators, input capture interruptions, etc. The motor control board also has a VNH2SP30: a full H-bridge motor driver from STMicroelectronics for direct motor control. This full H-bridge can operate up to 41 V and 30 A and provides multiple options to control direction, brake, speed and motor current monitoring. This motor control board has been used previously in [19] for testing and it is able to apply different PWM to three brushed DC motors, read the information provided by the magnetic encoders, and implement a PID control of the angular rotational velocity of brushed DC motors.

### 2.3. Input Capture Method

In general, there are three different approaches to measure the angular rotational velocity of a motor with the low-cost magnetic encoder used in this paper: counting the pulses generated, counting the time between two rising (or falling) edges, or counting the time between consecutive rising and falling (and falling and rising) edges. The alternative of counting the pulses generated by the encoder has a very low dynamic range because one complete rotation of the motor only generates three pulses. The alternative of counting the time between two rising (or falling) edges has a huge dynamic range and generates three angular rotational velocity estimates per rotation. The alternative of counting the time between a rising and falling edge and then between the consecutive falling and rising edges has a similar huge dynamic range but increases to six angular rotational velocity estimates per rotation.

In this paper, the measurement of the angular rotational velocity is performed with the input capture method applied to measure the time between consecutive rising and falling and falling and rising edges, which is able to provide six velocity estimates per motor rotation. In the case of ARM microcontrollers the input capture method can generate an interruption in the rising or falling edge of an input signal and automatically store the pulses counted by an internal timer. Then, the dynamic range of the input capture method is only limited by the number of bits of the internal timer counter used as a reference.

The microcontroller of the motor controller board has been configured to use the internal Timer 2, which has 32 bits, configured with the maximum allowed input clock: 84 MHz (internal operating frequency divided by two). This clock provides 84 × 10^6^ counts per second with a resolution of 11.9 ns per count so Timer 2 overflows (reach the maximum value of 4,294,967,295 counts and starts again with a zero value) every 51 s. The value of the Timer 2 counts are automatically stored in a register every time that the input capture method is activated and then the time lapse between two consecutive edge transitions can be obtained by computing the difference with the previous count stored.

## 3. The Rotary Encoder Misalignment Problem

**Figure 3:**
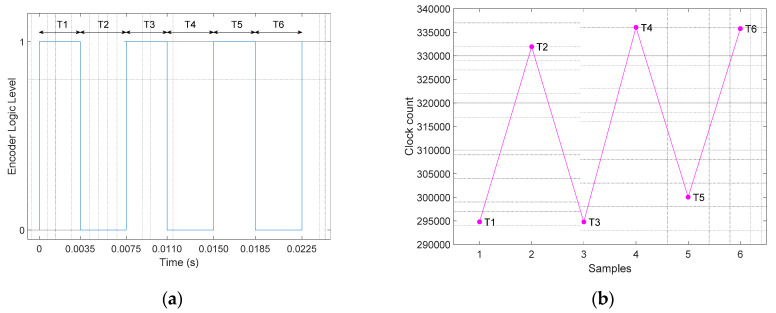
(**a**) Output signal from one Hall-effect sensor during one complete motor rotation. (**b**) Clock count values between consecutive rising and falling (and falling and rising) edges during one complete motor rotation.

Figure 3a shows an example of the sequence of pulses generated by the low-cost rotary encoder analyzed in this paper that generates three pulses in one complete rotation of the encoder. The input capture method provides the individual values of the time-lapse between consecutive edges *T*1, *T*2, *T*3, *T*4, *T*5, and *T*6. The assumption is that these time lapses measured in an ideal rotary encoder rotating at a constant angular rotational velocity will have the same value:

(1) $$T_{k}=T1=T2=T3=T4=T5=T6$$

The time required in this ideal case to complete one rotation *T* of the encoder is:

(2) $$T=T1+T2+T3+T4+T5+T6=6\cdot T_{MEAN}\;\;\;\;with\;\;\;\;T_{MEAN}=\frac{T}{6}\;$$

Then, the angular rotational velocity of rotary encoder can be computed as an average value representing a complete rotation

(3) $$\omega =\frac{2\pi }{T},\;or\;\;\;\;\omega =\frac{2\pi }{6\cdot T_{MEAN}}$$

or six times per rotation using:

(4) $$\omega_{k}=\frac{2\pi }{6\cdot T_{k}}$$

The possibility to obtain six estimates of the instantaneous rotational velocity measured by the rotary encoder in one rotation (instead of only one complete average value) is practical advantage in a closed loop control of a brushed DC motor of a mobile robot because the sampling time of the PID controller can operate with a faster sampling time. However, the possibility to obtain six estimates per rotation has some practical limitations because in a real encoder the time lapse sequence between consecutive edges *T*1, *T*2, *T*3, *T*4, *T*5, and *T*6 is affected by small structural misalignments and homogeneity differences in the magnetic field [20] applied to the Hall-effect sensors. Therefore, the accurate estimation of the angular rotational velocity of this magnetic rotary encoder requires the availability of six previous time-lapses. 

Figure 3b shows the values of the time-lapses measured in a real application case example. The time lapse values are represented by the clock count values directly measured by the input capture method defined previously. Figure 3b shows that the time-lapse between consecutive pulse edges has small differences or a ripple. In practice this ripple is usually avoided by filtering [21] or computing an average of the angular rotational velocity of the rotary encoder using Equation (3). This ripple needs to be specifically avoided because a fast PID controller interprets this measurement ripple as a real difference between the target and real motor velocity, generating unnecessary compensations and the final introduction of an oscillation in the evolution of the angular velocity of the motor. Furthermore, the effect of this ripple in the odometry of a mobile robot cannot be rejected with the application of calibration strategies because of the holonomic nature of the motion [22].

## 4. Improving the Measurement of the Instantaneous Angular Rotational Velocity

The hypothesis of this paper is that the information provided by a low-cost rotary encoder based on a rotating magnetic ring and fixed Hall-effect sensors can be processed and improved in order to obtain an accurate and precise estimation of the instantaneous angular rotational velocity. This hypothesis is based on the assumption that the magnetic rotary encoder must generate the output signal pulse defined by Equation (2) but the structural misalignment between the magnetic field of the ring and the Hall-effect sensors originates different time-length measurements with:

(5) $$\begin{array}{l} T1=T_{MEAN}\cdot \left(1+\epsilon_{1}\right)\\ T2=T_{MEAN}\cdot \left(1-\epsilon_{1}+\epsilon_{2}\right)\\ T3=T_{MEAN}\cdot \left(1-\epsilon_{2}+\epsilon_{3}\right)\\ T4=T_{MEAN}\cdot \left(1-\epsilon_{3}+\epsilon_{4}\right)\\ T5=T_{MEAN}\cdot \left(1-\epsilon_{4}+\epsilon_{5}\right)\\ T6=T_{MEAN}\cdot \left(1-\epsilon_{5}\right) \end{array}$$

where *ε_k_* represents the relatively small variation of the time-location of the edges in the encoder output signal. Then, these structural errors can be corrected by obtaining and applying the misalignment correction coefficients *Mk* of the rotary encoder:

(6) $$\begin{array}{l} T_{MEAN}=\frac{T1}{\left(1+\epsilon_{1}\right)}=\frac{T1}{M1}\\ T_{MEAN}=\frac{T2}{\left(1-\epsilon_{1}+\epsilon_{2}\right)}=\frac{T2}{M2}\\ T_{MEAN}=\frac{T3}{\left(1-\epsilon_{2}+\epsilon_{3}\right)}=\frac{T3}{M3}\\ T_{MEAN}=\frac{T4}{\left(1-\epsilon_{3}+\epsilon_{4}\right)}=\frac{T4}{M4}\\ T_{MEAN}=\frac{T5}{\left(1-\epsilon_{4}+\epsilon_{5}\right)}=\frac{T5}{M5}\\ T_{MEAN}=\frac{T6}{\left(1-\epsilon_{5}\right)}=\frac{T6}{M6} \end{array}$$

**Figure 4:**
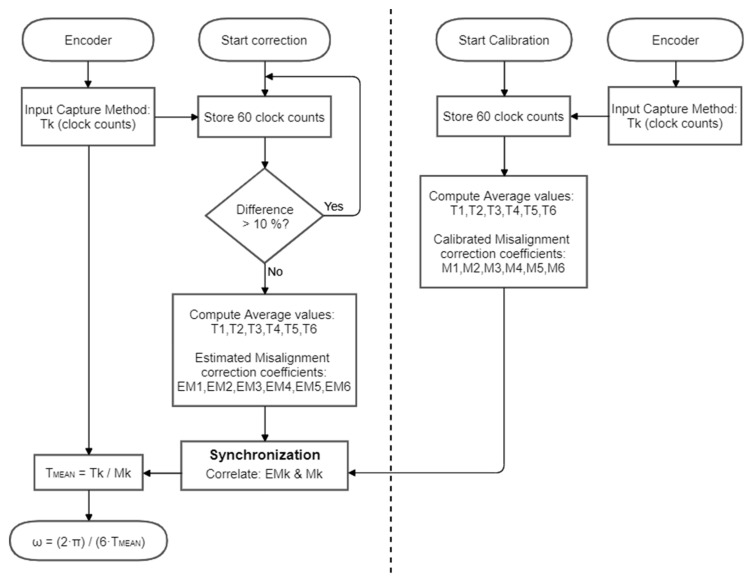
Flowchart diagram of the calibration procedure (**right**) and correction procedure (**left**) in order to obtain a new accurate estimation of the instantaneous angular rotational velocity of the rotary encoder.

Figure 4 shows the flowchart or the procedures proposed to obtain the misalignment correction coefficients *Mk* in order to improve the measurement of the angular rotational velocity. There are two different procedures: (1) a calibration procedure that automatically analyze the output signal of the rotary encoder in order to obtain the misalignment correction coefficients that can compensate the differences in the length of the rising and falling edge transitions, and (2) a correction procedure that synchronizes the application of the misalignment correction coefficients with the pulse information provided by the rotary encoder. The application of this correction procedure will provide a new accurate estimation of the instantaneous angular rotational velocity of the encoder on every new edge of the encoder signal.

### 4.1. Calibration Procedure

**Figure 5:**
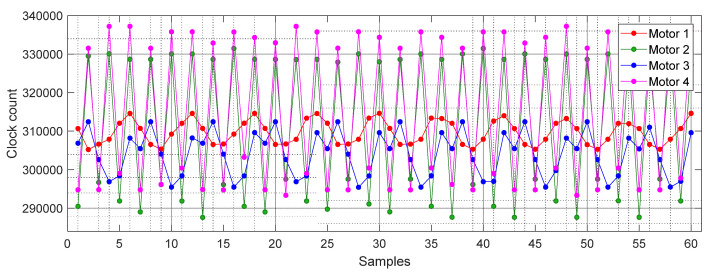
Time measured between rising and falling edge transitions of the encoder signal expressed in clock counts. Sequence obtained from four different compact geared brushed DC motor including a low-cost magnetic rotary encoder attached to the motor.

Figure 4 shows the flowchart diagram of the calibration procedure proposed to obtain the misalignment correction coefficients of the magnetic rotary encoder. The procedure is based on analyzing the output pulse signal when the rotary encoder rotates at a constant angular velocity. This effect has been achieved by applying a fixed PWM of 80% (or in the range between 70–100%) to the brushed DC motor. Figure 5 shows the time-length sequence obtained with four different compact brushed DC motors rotating at stationary angular velocity. The value of the pulse lengths shown are directly expressed in clock count values obtained from Timer 2. The values shown correspond to 10 complete motor turns and they have 60 samples with a periodicity of 6 samples. Figure 5 shows that different magnetic encoders from different motorizations generate different asymmetric periodic time-length sequences.

**Figure 6:**
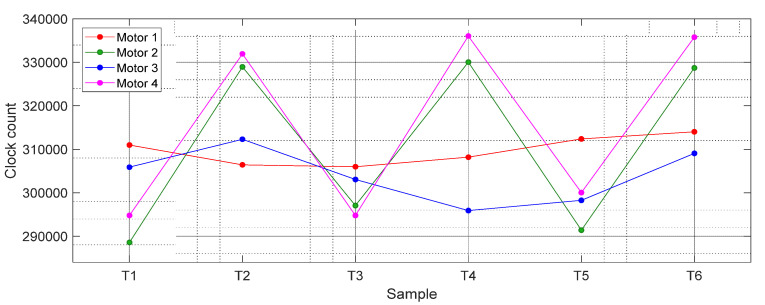
Average clock count values obtained in the four different motorizations analyzed.

**Figure 7:**
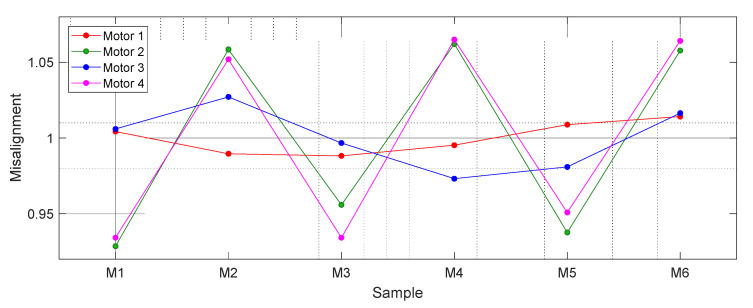
Misalignment correction coefficients corresponding to the rotary encoders analyzed.

Figure 6 shows the average values of *T*1, *T*2, *T*3, *T*4, *T*5, and *T*6 from the data shown in Figure 5 and Figure 7 shows the value of the misalignment correction coefficients required to obtain the real angular rotational velocity of the motorizations. Results show that Motor 4 (magenta) and Motor 2 (green) cases showed the highest variations (misalignments), while the Motor 1 case showed the lowest variations in the clocks counted.

The advantage of the application of this misalignment correction coefficients is that a magnetic encoder with six poles (generating 3 pulses per rotation) provides six corrected and precise instantaneous angular rotational velocity estimates *T* (Equation (6)) per motor rotation. Just as a reference, the output signal generated by the encoder when the brushed DC motor rotates at its minimum speed (around 160 rpm) will generate 16 new angular velocity estimates per second so, in this case, the PID of the motor can operate with a periodicity of up to 16 Hz (or 62.5 ms). Alternatively, the output signal generated by the encoder when the brushed DC motor rotates at its maximum speed (around 3.800 rpm) will generate 380 new angular velocity estimates per second so, in this case, the PID of the motor can operate with a periodicity of up 380 Hz (or 2.63 ms).

**Figure 8:**
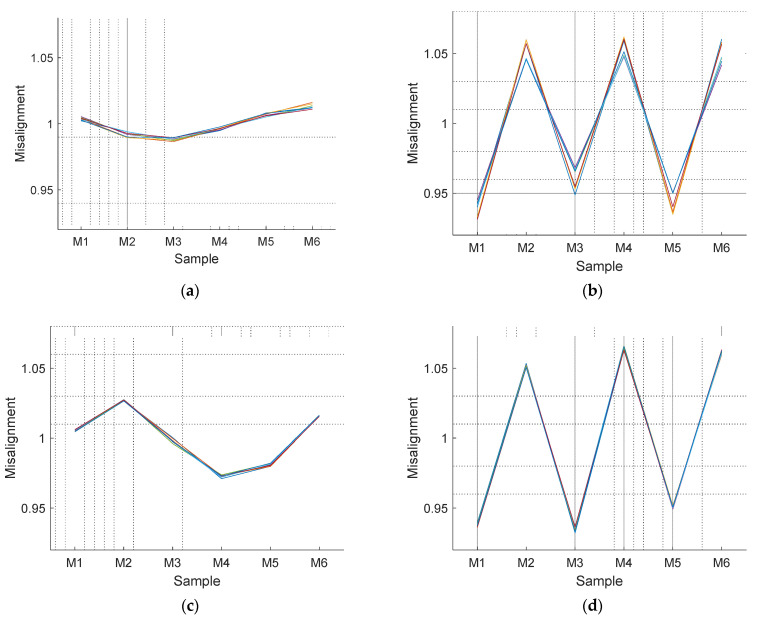
Representation of the misalignment correction coefficients obtained for duty cycles: 30, 40, 50, 60, 70, 80, 90, and 100% for: (**a**) Motor 1, (**b**) Motor 2, (**c**) Motor 3, and (**d**) Motor 4.

The next step is the verification if the misalignment correction coefficients have a dependence with the angular rotational velocity of the motor. Figure 8 shows the misalignment correction coefficients obtained after repeating the previous experiment with the following PWM values (and approximate values of the rpm achieved when the motor rotates at constant velocity): 30% (954 rpm), 40% (1354 rpm), 50% (1747 rpm), 60% (2115 rpm), 70% (2520 rpm), 80% (2873 rpm), 90% (3637 rpm), and 100% (3806 rpm). The misalignment correction coefficients shown in Figure 8 show no dependence with the angular velocity, confirming the hypothesis of the structural misalignments of the rotary encoders.

Finally, Table 1 summarizes the values of the misalignment correction coefficients of the four motorizations analyzed in this paper. These specific and individual values can be used to improve the measurement of the instantaneous angular rotational velocity and the closed loop control of each motorization.

### 4.2. Correction Procedure

Figure 4 also shows the flowchart diagram of the correction procedure proposed to apply the misalignment correction coefficients of the magnetic rotary encoder. The correction procedure has been designed to be used when the motorization is in normal use, without requiring a specific control procedure. The assumption is that the motor controller board that drives the motorization has access to the misalignment correction coefficients (*M*1, *M*2, *M*3, *M*4, *M*5, *M*6) obtained during a previous calibration procedure of the brushed DC motor and rotary encoder.

The correction procedure by itself consists of applying the correct misalignment correction coefficient to the current time-lapse measured in clock counts by the input capture method. The procedure proposed to synchronize the misalignment correction coefficient is based on obtaining an estimate of the misalignment correction coefficient and has the following internal steps. (1) Similarly, as in the calibration procedure, the motor controller board reads and stores 60 time-lapse values (Figure 5), then computes the average value and the maximum difference of the sequence relative to this average value. If this maximum difference is higher than 10% (probably because the motor is still accelerating) then the stored values are discarded, and this first step is repeated until the rotary encoder provides stationary measurements. (2) The values stored are averaged with a periodicity of 6 in order to obtain an estimate of the six characteristic time-lapse values *T*1, *T*2, *T*3, *T*4, *T*5, and *T*6, and an additional cumulated time-length T corresponding to a complete rotation. Then, these values are used to obtain an estimate of the misalignment correction coefficients EM1, EM2, EM3, EM4, EM5, and EM6. The assumption is that these estimates are a noisy estimate of the misalignment correction coefficients because the motorization is operating in real charge and closed-loop conditions. (3) Finally, the estimated misalignment correction coefficients are correlated with the calibration values using a circular sequence comparison in order to find the best match and synchronize the application of the misalignment correction coefficients. (4) After this synchronization, every new time-lapse between consecutive edges is corrected in order to obtain a precise estimation of the angular rotational velocity of the encoder.

**Figure 9:**
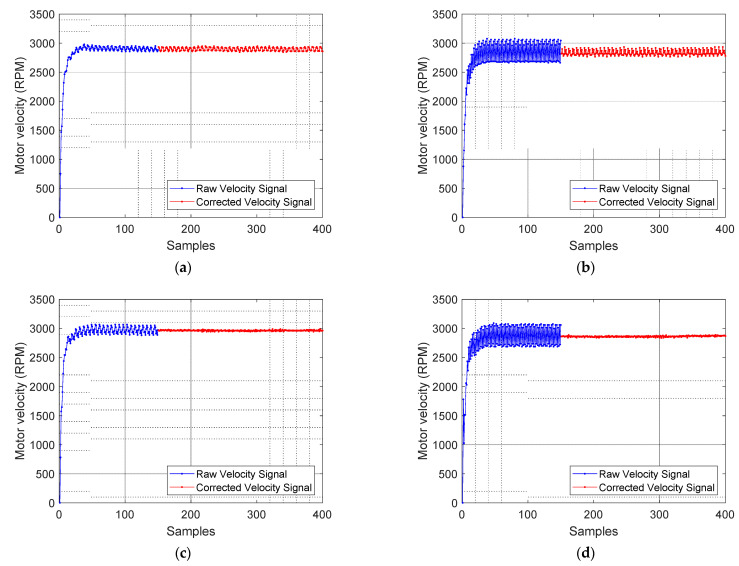
Effect of the application of the correction procedure proposed to improve the estimation of the angular velocity of (**a**) Motor 1, (**b**) Motor 2, (**c**) Motor 3, and (**d**) Motor 4. The blue color depicts the raw instantaneous angular rotational velocity of the motorization and the red color depicts the corrected rotational velocity.

Figure 9 shows the validation experiments of the correction procedure applied to the four compact motorizations analyzed in this paper. In each validation experiment the motor controller board applies a PWM with a fixed duty cycle of 80%. The synchronization of the misalignment correction coefficients only requires a constant angular rotational velocity during 60 samples, but in this validation experiment the application of the correction procedure has been delayed until the 150th sample count in order to evidence the improvement. The ripple reduction obtained was 4.93% for Motor 1 (Figure 8a), 59.43% for Motor 2 (Figure 8b), 76.49% for Motor 3 (Figure 8c), and 86.75% for Motor 4 (Figure 8d) which was the motor having the most misaligned magnetic encoder. The application of this correction procedure in a closed-loop angular rotational velocity control produces the same improvements and simplifies the control of the motorization.

As a non-optimal solution, the estimated misalignment correction coefficients obtained during step 2 of the correction procedure can be applied directly to correct the counts measured by the input capture method. However, then, the estimated misalignment correction coefficients may be influenced by the closed-loop control applied to the motorization.

## 5. Discussion and Conclusions

This paper proposes a procedure to improve the instantaneous angular velocity measured with the low-cost magnetic rotary encoder that is usually used attached to brushed DC motors. This magnetic rotary encoder is based on a rotating magnetic ring and two fixed Hall-effect sensors that provide a digital output pulse-length proportional to the rotation. This paper has validated the hypothesis that the misalignments between the magnet and Hall-effect sensors are the cause of the error or ripple in the estimated angular rotational velocity provided by the encoder.

In general, the problem originated by magnetic misalignments has been studied in deep in the case of brushless DC (BLDC) motors because this internal configuration affects the motor performances [8,9]. However, this structural effect is less evident in the case of an external low-cost magnetic rotary encoder attached to a brushed DC motor and has not been previously analyzed.

The procedure proposed to improve the estimation of the instantaneous angular rotational velocity provided by a low-cost magnetic rotary encoder is based on a calibration procedure and on a correction procedure. The calibration procedure provides the misalignment correction coefficients under an open-loop control of the motor and the rotary encoder. Then, the correction procedure is used to synchronize the application of the misalignment correction coefficients with the pulse-length information obtained from the rotary encoder. The analysis of four motorization cases using a low-cost magnetic encoder has found misalignment correction coefficients in a range between 1.0132 and 0.9882, 1.0542 and 0.9365, 1.0257 and 0.9728, and 1.0652 and 0.9336. These correction values may seem very small but the instantaneous estimation of the angular rotational velocity of the encoder multiplies by 6 the time-length measured from the encoder, so any misalignment improvement will have a large impact in the final value estimated. In concordance with this expectation, the application of the correction procedure has reduced the amplitude of the ripple or variation of the instantaneous angular rotational velocity in 4.93%, 59.43%, 76.49%, and 86.75%, respectively. This reduction improves the precision of the estimation of the angular rotational velocity of the typical motorizations used in mobile robotics that are based on a low-cost magnetic rotary encoder attached to a geared brushed DC motor.

Future work will be focused on evaluating the impact of this improvement in the control and odometry of different mobile robots using compact motorizations based on geared brushed DC motors and low-cost magnetic encoders.

## Figures and Tables

**Table 1:** Misalignment correction coefficients of the four motorizations analyzed.

Motor	Misalignment Correction Coefficients						
	*M*1	*M*2	*M*3	*M*4	*M*5	*M*6	
1	1.0037	0.9919	0.9882	0.9963	1.0068		1.0132
2	0.9365	1.0537	0.9636	1.0542	0.9426		1.0494
3	1.0060	1.0257	0.9994	0.9728	0.9799		1.0162
4	0.9377	1.0519	0.9336	1.0652	0.9512		1.0606

## Data Availability

Data sharing not applicable.
